# Clinical value of bioelectrical properties of cancerous tissue in advanced epithelial ovarian cancer patients

**DOI:** 10.1038/s41598-018-32720-8

**Published:** 2018-10-02

**Authors:** Paula Cunnea, Tommy Gorgy, Konstantinos Petkos, Sally A.N. Gowers, Haonan Lu, Cristina Morera, Wen Wu, Phillip Lawton, Katherine Nixon, Chi Leng Leong, Flavia Sorbi, Lavinia Domenici, Andrew Paterson, Ed Curry, Hani Gabra, Martyn G. Boutelle, Emmanuel M. Drakakis, Christina Fotopoulou

**Affiliations:** 1 Department of Surgery and Cancer, Imperial College, London, UK; 2 Department of Bioengineering, Imperial College, London, UK; 3 Department of Biomedical, Experimental and Clinical Sciences, University of Florence, Florence, Italy; 4 Department of Obstetrics, Gynecology and Urologic Sciences, University “Sapienza” of Rome, Rome, Italy; 5 Early Clinical Development, IMED Biotech Unit, AstraZeneca, Cambridge, UK

## Abstract

Currently, there are no valid pre-operatively established biomarkers or algorithms that can accurately predict surgical and clinical outcome for patients with advanced epithelial ovarian cancer (EOC). In this study, we suggest that profiling of tumour parameters such as bioelectrical-potential and metabolites, detectable by electronic sensors, could facilitate the future development of devices to better monitor disease and predict surgical and treatment outcomes. Biopotential was recorded, using a potentiometric measurement system, in *ex vivo* paired non-cancerous and cancerous omental tissues from advanced stage EOC (n = 36), and lysates collected for metabolite measurement by microdialysis. Consistently different biopotential values were detected in cancerous tissue versus non-cancerous tissue across all cases (p < 0.001). High tumour biopotential levels correlated with advanced tumour stage (p = 0.048) and tumour load, and negatively correlated with stroma. Within our EOC cohort and specifically the high-grade serous subtype, low biopotential levels associated with poorer progression-free survival (p = 0.0179, p = 0.0143 respectively). Changes in biopotential levels significantly correlated with common apoptosis related pathways. Lactate and glucose levels measured in paired tissues showed significantly higher lactate/glucose ratio in tissues with low biopotential (p < 0.01, n = 12). Our study proposes the feasibility of biopotential and metabolite monitoring as a biomarker modality profiling EOC to predict surgical and clinical outcomes.

## Introduction

Epithelial ovarian cancer (EOC) is a heterogeneous disease with a high risk of relapse and development of platinum resistance conferring a poor prognosis^1^. The cornerstone of treatment is maximal-effort surgical cytoreduction combined with a cytotoxic and targeted systemic approach^2^. In recent years, ultra-radical surgery has increasingly become more commonplace with multivisceral resection techniques reaching beyond standard surgery and extending beyond the pelvis and even the abdomen to the thoracic cavity^3^. This ultra-radical approach requires not only a high institutional effort overall and highly specialized surgical expertise, but also maximal patient physical resources. However, despite a maximal effort approach, outcomes vary broadly with 20% of patients experiencing an early relapse with a less favourable outcome despite their initial tumour-free surgery^4^. With the morbidity and mortality of ultra-radical procedures being on average as high as 20% and 1–3% respectively^2^, the National Institute for Health and Care Excellence (NICE) UK in 2013 published interventional procedure guidance about the use and value of ultra-radical surgery for advanced ovarian cancer and recommended its application only in carefully selected patients in an effort to reduce unnecessary iatrogenic morbidity^5^. To date, there are no valid pre-operatively established algorithms or biomarkers that can reliably inform on surgical and clinical outcome in order to adapt the extent and radicality of treatment to each patient’s needs and tumour biology.

In an attempt to profile patient tumour biology and predict patient clinical outcome and surgical success, we exploited the basic property of every cell to develop electrical biopotential across its membrane due to the presence of different ion concentrations between the intracellular and extracellular space. Interestingly, a cell’s behaviour is controlled not only by its own resting potential but also by the potential of its neighbouring cells^6^. Moreover, the role of endogenous voltage potentials and bioelectric gradients in the dysregulation of cell interactions leading to cancer has been highlighted recently^7,8^. Furthermore, ion channels have been suggested to be involved in cancer development and progression, from the initial proliferation process to metastasis, and in particular in multidrug resistance, which is associated with the treatment of ovarian cancer^9^. It has been shown that cancerous cells produce a different bioelectrical potential across the cell membrane than normal cells in different cancer types, including breast cancer^10–13^.

**Figure 1:**
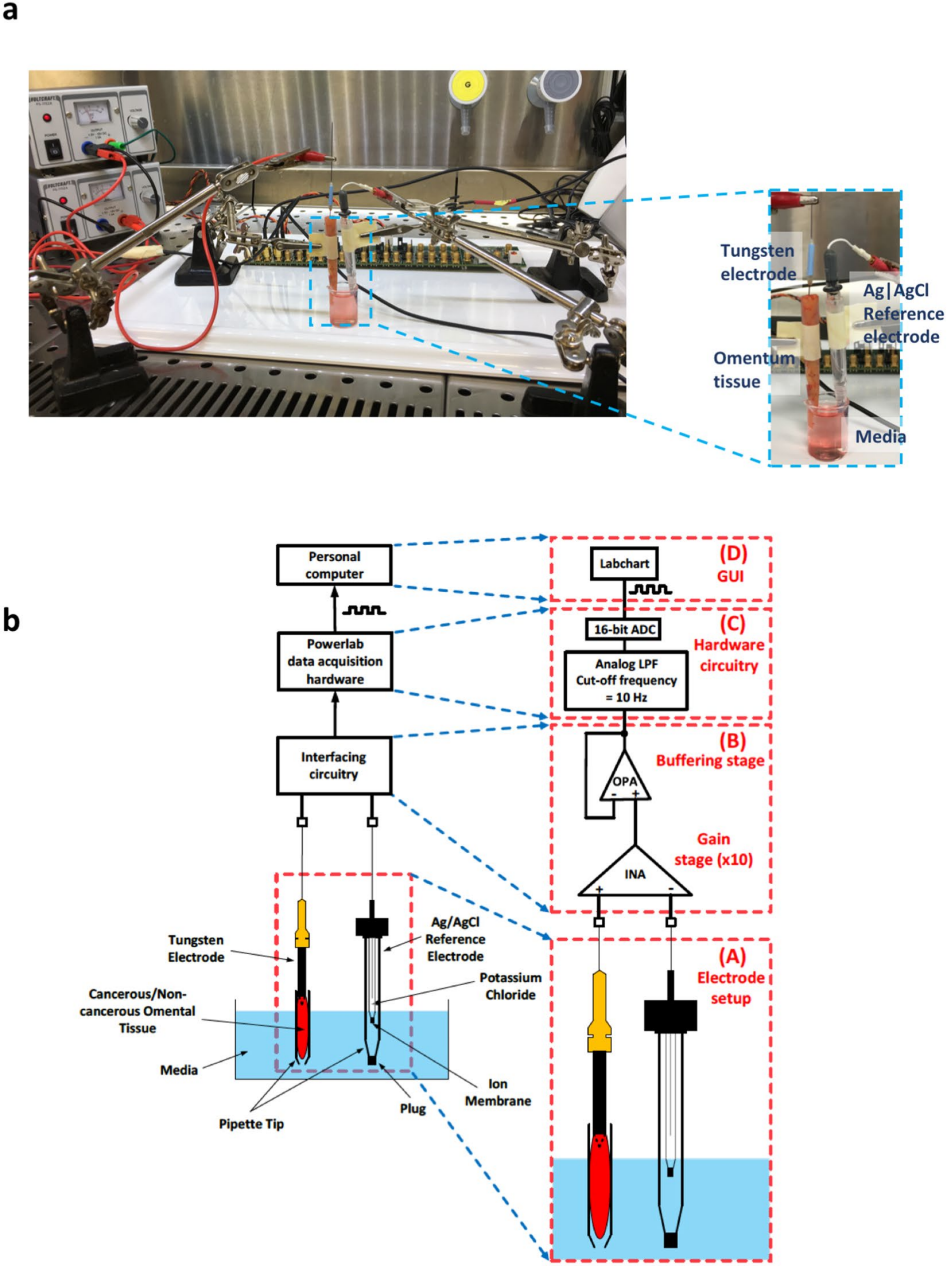
Laboratory set up for recording of biopotential readings. (**a**) Experimental laboratory set up for recording of biopotential measurements in a sterile laminar flow cabinet, measurements are recorded using Labchart software on a laptop set up outside the laminar flow cabinet. Inset shows the tungsten working electrode inserted in a piece of non-cancerous omentum tissue and partly submerged in media. The Ag|AgCl reference electrode is also placed in a 1 ml pipette tip which is partly submerged in the same media beaker. (**b**) Developed methodology for recording of tissue biopotential readings. The analog voltage difference, present between a tungsten electrode that pierces the human tissue and a Ag|AgCl reference electrode submerged in cell culture medium solution, is recorded by means of an interfacing circuitry. The output of the interfacing circuitry is processed by a commercially available data acquisition system (Powerlab data acquisition hardware) and is depicted on the computer screen. (A) Detailed view of the electrochemical cell. Both the Ag|AgCl reference electrode and the tissue sample are in contact with cell culture medium solution. The tip of the tungsten electrode (dashed line) pierces the cancerous/non-cancerous tissue sample but does not make contact with the medium solution. This ensures that the voltage measurement is taken from the tissue sample and not from the medium solution. (B) Detailed view of the interfacing circuitry. It consists of a high-performance instrumentation amplifier (INA), which amplifies the voltage difference between the tungsten and the Ag|AgCl double junction reference electrode, and an operational amplifier (OPA) connected as a voltage follower, which acts as a buffering stage between the interfacing circuitry and the following stage (Powerlab data acquisition hardware). (C) Detailed view of the commercially available Powerlab data acquisition hardware. The amplified signal coming from the interfacing circuitry is low-pass filtered (LPF) with the cut-off frequency set at 10 Hz. Next, the filtered signal is digitized by a 16-bit analog-to-digital converter (ADC). (D) The digital signal is depicted on the computer screen using the graphical user interface (GUI) offered by the Powerlab system (Labchart).

With the help of newly developed methodology^14,15^, we established a feasibility study to determine the practicality of using biopotential as a predictive biomarker of surgical and clinical outcome after radical cytoreductive surgery for advanced primary or relapsed EOC. Our method enables the measurement of potential differences between paired macroscopically healthy and cancerous tissue samples by means of a combination of a Ag|AgCl electrode, a tungsten electrode and interfacing electronics, which allows for the recording of the biopotential present between the Ag|AgCl electrode and the tissue sample pierced by the tip of the tungsten electrode. Figure 1a illustrates the experimental laboratory setup used for the tissue biopotential measurements while the method itself and its constituent components are detailed in Fig. 1b.

We measured biopotential in a cohort of *ex vivo* paired cancerous and non-cancerous omentum tissues excised during radical debulking surgeries for advanced EOC, and report associations between biopotential and tumour stage, and patient progression-free survival. As cancer cell metabolism is known to be anaerobic, in some paired samples in addition to monitoring biopotential, we also monitored tissue metabolism using microdialysis sampling and online measurement of glucose and lactate using biosensors^16,17^. Our overall objective is to generate a usable, validated biopotential and metabolic signature that could be applied at the onset of treatment efforts to predict clinical outcome in terms of early relapse and onset of platinum resistant disease and fulfil the highly unmet need of personalisation of surgical treatment in advanced ovarian cancer.

## Results

### Biopotential can be consistently measured in *ex vivo* tissue and differs between cancerous and non-cancerous omentum tissue

We developed a measurement system^14^ to facilitate reliable stable detection of resting biopotential in *ex vivo* cancerous and non-cancerous omentum tissue. An image of the working laboratory measurement set up is displayed in Fig. 1a. The larger magnification image shows the tungsten working electrode inserted into a non-cancerous omentum tissue, both the tissue with tungsten electrode and Ag|AgCl reference electrode are partly submerged in the same media solution, and a measurement is recorded on Labchart software. Figure 1b illustrates in detail the methodology developed^14,15^ and the main components of our tissue measurement system for recording tissue biopotential measurements. The biopotential present between a tungsten electrode which pierces the human tissue sample and a Ag|AgCl reference electrode is recorded by means of appropriate interfacing circuitry whose output is processed by a commercially available data acquisition system and is subsequently visualised onto a computer screen. Both the tissue sample and the reference electrode are in contact with cell culture media solution with the tip of the tungsten electrode piercing the tissue sample without making contact with the media solution. Sub-panels (A)-(D) of Fig. 1b offer functional block representations of the main components constituting our measurement method.

**Figure 2:**
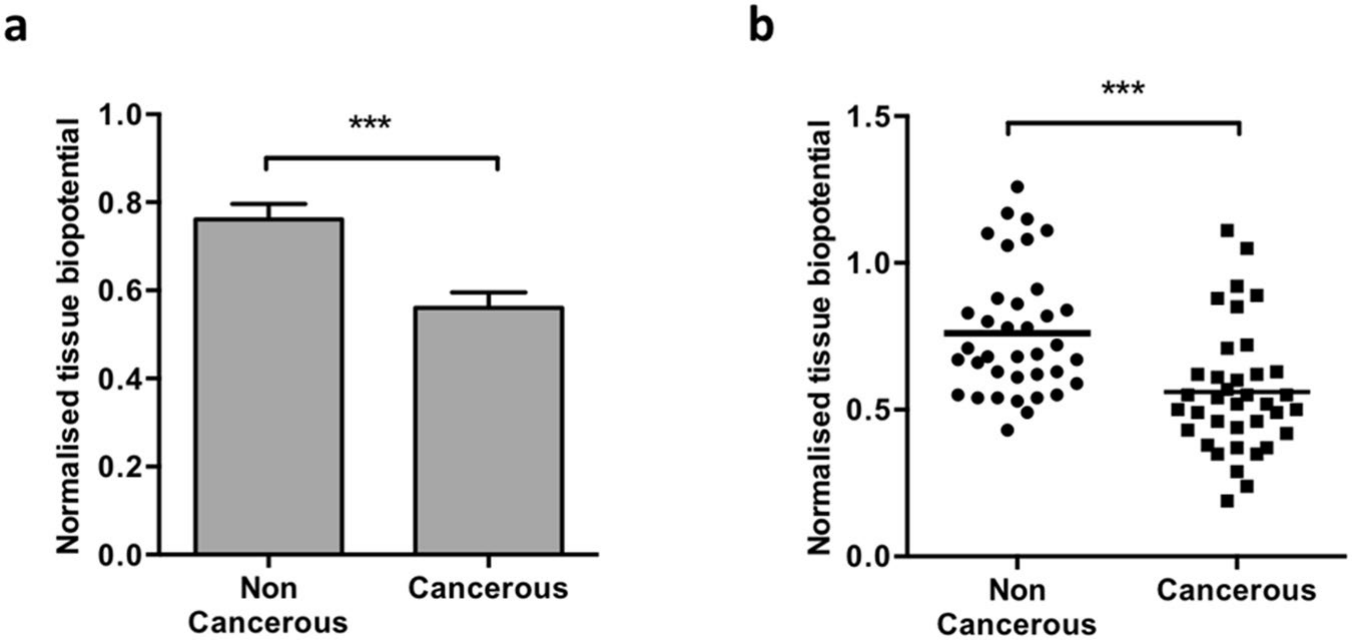
Biopotential levels differ between paired cancerous and non-cancerous tissues. (**a**) Biopotential data captured in the laboratory 45–120 min post removal on excised, paired omentum samples with macroscopically non-cancerous and cancerous appearance in different areas of the tissues. All biopotential readings were measured in triplicate and normalized to a reference media reading. Across the patient series, lower biopotential readings were observed for all cancerous samples compared to its paired non-cancerous counterpart (p < 0.001, n = 36). (**b**) A range of biopotential readings were collected across the patient series (each dot represents the mean of each tissue’s biopotential) for all tissues collected, consistently cancerous tissue had a lower biopotential than its non-cancerous pair (p < 0.001, n = 36).

The resting biopotential was measured in triplicate in different regions of paired macroscopically non-cancerous and cancerous omentum tissues excised from patients undergoing maximal effort primary cytoreductive surgery for advanced epithelial ovarian cancer (n = 36). We consistently observed a significantly lower biopotential measurement in the cancerous tissue compared to the paired non-cancerous tissue (Fig. 2a, p < 0.001). The trend of lower cancerous biopotential values versus non-cancerous readings was consistent in all paired tissues, over all cases, despite a large range of biopotential values recorded (Fig. 2b, p < 0.001). Additionally, to determine whether the size of the tissue measured had any influence on biopotential levels, excised cancerous tissue samples were measured for biopotential, and dissected into smaller sizes (width or depth) while retaining the working tungsten electrode in the same position within the tissues to record biopotential following each dissection. No change in biopotential was observed in any of the tissues dissected (Supplementary Figure S1).

### Biopotential levels associate with tissue cellularity

**Figure 3:**
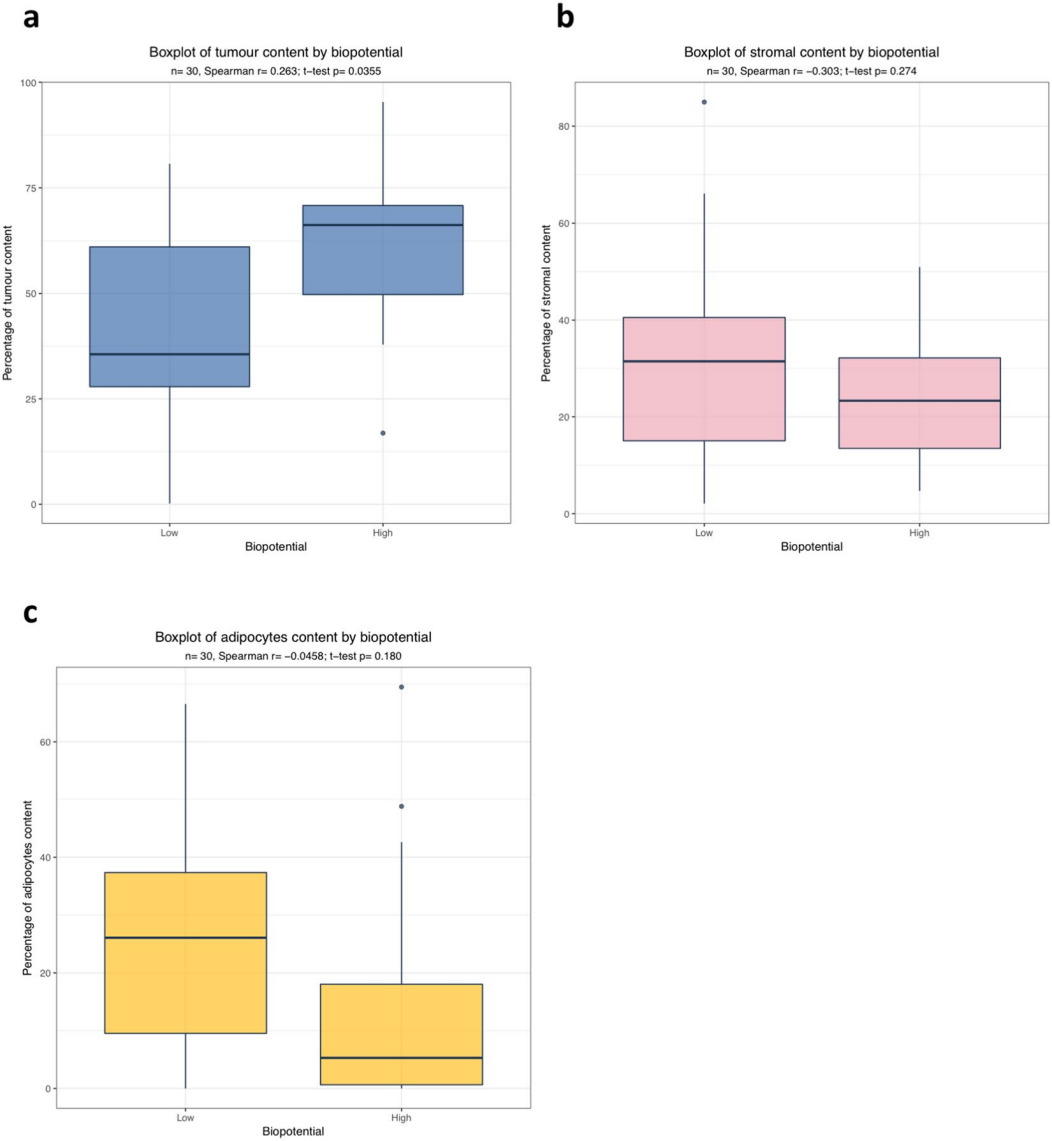
Potential biological correlations with biopotential readings in advanced ovarian cancer. Three different sites in the cancerous omentum from ten EOC cases were analysed. (**a**) Box plot representing the positive correlation between tumour content and biopotential levels, stratified at normalized median biopotential levels into low (below the median) versus high (above the median). High biopotential levels significantly correlated with increased tumour load (p = 0.0355) (**b**) Box plot showing that increased stromal content negatively correlates with low biopotential. (**c**) Adipocytes content and biopotential showed a trend with increased adipocyte load correlating with low biopotential. The correlations were measured by Spearman’s correlation coefficient and t-test.

Immunohistochemistry for the tumour markers Pax8 and WT1, commonly associated with EOC and HGSOC^18^, was performed to determine the tumour cell load in tissues and demonstrated the presence of micro-metastasis of tumour cells in some of the macroscopically non-cancerous tissues measured (Supplementary Figure S2). Biopotential readings were correlated with a tumour, stromal and adipose tissue score for the cancerous tissues (Fig. 3) and non-cancerous tissues (Supplementary Figure S3). For the cancerous tissue, a positive correlation between increasing tumour load and biopotential, and a negative association between higher biopotential levels and the amount of stroma present was detected. When stratifying at median biopotential levels for the cancerous tissues, we observed biopotential levels above the median biopotential associated with higher tumour content in the tumour tissues (Fig. 3a) and biopotential levels below the median associated with a higher percentage of stroma in the tumour tissues (Fig. 3b), with a similar trend observed between lower biopotential levels and adipocyte content in the tumour tissues (Fig. 3c). Little correlation was observed between tumour, stroma and adipocyte content in the macroscopically non-cancerous tissues (Supplementary Figure S3).

Furthermore, to establish if cell density influenced biopotential levels, we suspended increasing numbers of immortalised cells (SKOV3) in a 3D collagen matrix model *in vitro* and measured the biopotential of each 3D model. We observed decreasing levels of biopotential in the collagen matrix modified with increasing numbers of cells (Supplementary Figure S4a). Moreover, using different densities of agarose as an *in vitro* tissue surrogate to examine the influence of matrix (tissue) stiffness on biopotential, we determined that biopotential levels decreased in a series of gel matrices with increasing density (Supplementary Figure S4b). Thus, showing that biopotential levels can be influenced by cell type, cell number and matrix stiffness.

### Biopotential levels in omentum tumours associate with late stage and poor clinical outcome

**Figure 4:**
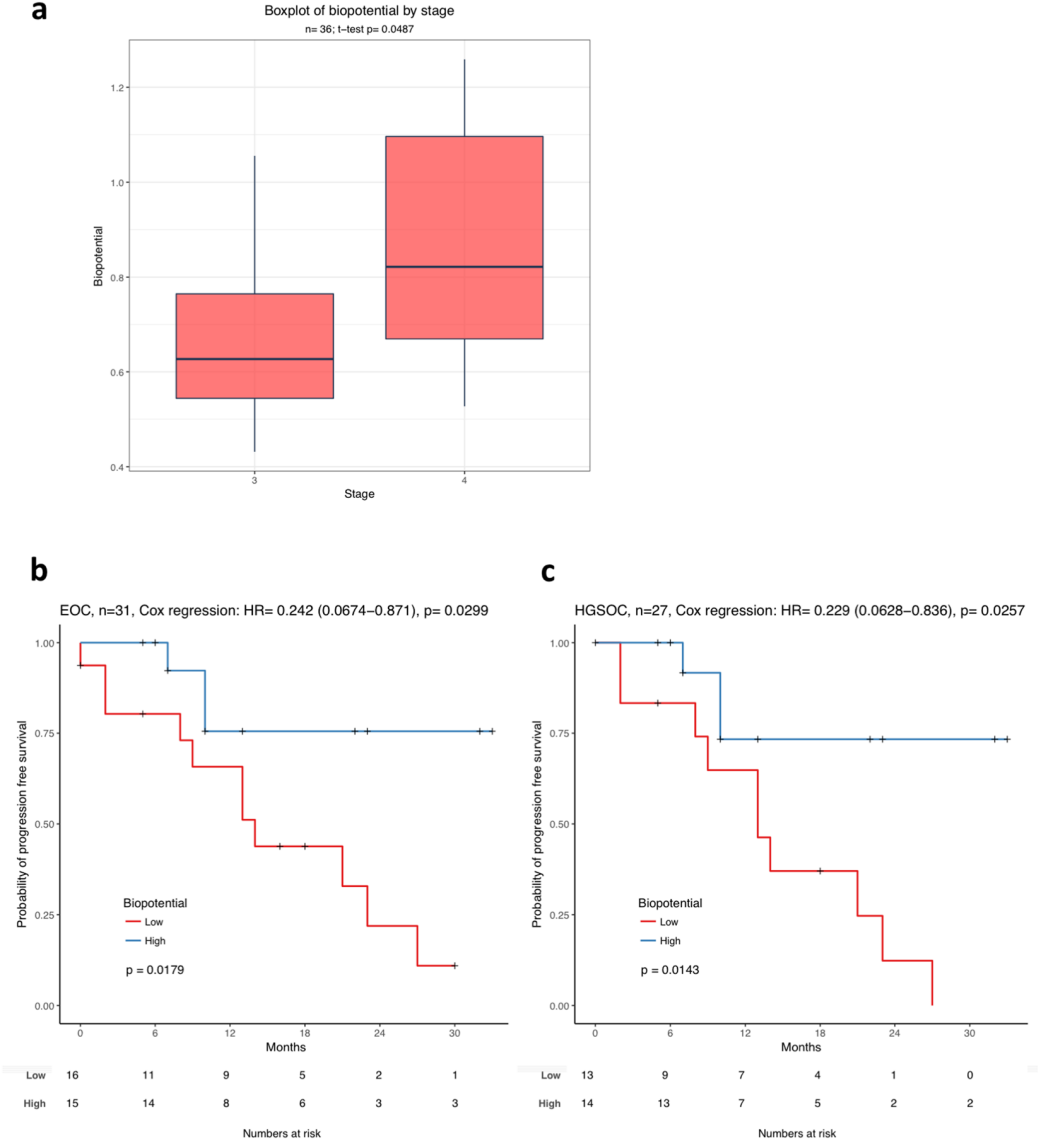
Biopotential readings correlate with tumour stage and poor progression free survival. (**a**) Biopotential readings of all advanced ovarian cancer cases at Stage III (n = 18) and Stage IV (n = 18) shows that higher biopotential levels associate with late stage. The p-value was determined by Welch two Sample t-test (p = 0.0487). (**b**) Kaplan-Meier plot representing the biopotential association with PFS in ovarian cancer cases of all subtypes (n = 31). (**c**) Biopotential association with PFS in high grade serous ovarian cancer (n = 27). In both Kaplan-Meier analysis, the patients were dichotomised at median biopotential and formed the biopotential high group (red) and the biopotential low group (blue). Within the cohort of patients with biopotential below the median, it was a 2:1 ratio of patients with Stage III versus Stage IV EOC disease. The p-value was given by log-rank test. Additionally, hazard ratio (HR), 95% confidence interval and the corresponding p-value was given by Cox regression.

All biopotential measurements were correlated with the clinical details and outcome of each patient (Table 1). We observed a significant correlation of high biopotential readings in the cancerous omentum tissues associated with advanced tumour stage (Fig. 4a, p = 0.04873). Furthermore, we examined whether there was any correlation of biopotential levels measured in the cancerous tissues with progression-free survival (PFS) for the entire cohort of patients (Fig. 4b) and for patients with only the HGSOC subtype (Fig. 4c). When biopotential levels are stratified at the median normalised cancerous biopotential, biopotential levels below the median were associated with a poorer PFS for all EOC histotypes and HGSOC only cases (p = 0.0179 and p = 0.0143 respectively).

**Table 1:** Clinical and histological details of all patients enrolled in the study (n = 36).

*Patient information*	*N* = 36
Patient age	
Median	64
Range	32–84
Death	
No	31
Yes	5
Recurrence	
No	22
Yes	11
Histology	
Low grade serous	2
High grade serous	32
Clear cell	2
FIGO Stage	
III	18
IV	18
Residual disease	
Tumour free	31
<0.5 cm	4
>0.5 cm	0
1-2 cm	0
>2 cm	1

### Changes in tissue biopotential levels correlate with apoptosis pathways

Reverse phase protein array (RPPA) proteomic profiling was performed on paired non-cancerous and cancerous tissues from 10 patients with HGSOC within our cohort to assess the effect of differing tissue biopotential levels on protein signalling. These patients were selected based on the extreme differences in biopotential recorded between their cancerous and non-cancerous tissues. Common pathways that were statistically significant following linear regression analysis were apoptosis related pathways (Table 2). When analysing correlation statistics in biopotential changes between non-cancerous and cancerous samples and protein expression, the cell cycle protein CDK1 was the only statistically significantly altered protein observed (Table 3).

**Table 2:** Pathway analysis of the log-fold change in protein expression changes with normalised biopotential changes.

Pathway Identifier	P Value	Adjusted P Value
Intrinsic Pathway for Apoptosis	9.8E-07	0.0011
Activation of BH3-only proteins	2.4E-05	0.014
Programmed Cell Death	6.6E-05	0.017
Apoptotic signaling in response to DNA damage	8.0E-05	0.017
Apoptosis	9.0E-05	0.017
Activation of BAD and translocation to mitochondria	1.1E-04	0.017
TCF dependent signaling in response to WNT	1.2E-04	0.017
Glucagon signaling pathway - Homo sapiens (human)	1.9E-04	0.024
Metformin Pathway, Pharmacodynamic	3.8E-04	0.039
TNF alpha Signaling Pathway	4.0E-04	0.039
TWEAK Signaling Pathway	4.1E-04	0.039
Corticotropin-releasing hormone	4.6E-04	0.040

Pathway enrichment analysis was performed to determine pathways that are significantly altered when comparing protein expression changes between cancerous and non-cancerous samples and biopotential. Correlations between these factors were ranked upon the gradient of the trend observed. All significantly enriched pathways are shown, with apoptosis related pathways being frequently represented.

**Table 3:** Change in normalised biopotential vs protein log fold change between cancerous and non-cancerous samples.

Protein Probe	logFC	P Value	Adjusted P Value
CDK1	10.08	9.3E-05	0.028
INPP4b	−2.08	6.7E-04	0.064
FAK pY397	−2.85	8.7E-04	0.064
PKM2	9.05	1.1E-03	0.064
EMA	12.70	1.2E-03	0.064
PAICS	2.93	1.9E-03	0.064
CD31	−2.79	1.9E-03	0.064
Bcl xL	4.25	2.2E-03	0.064
Bcl2	−3.18	2.3E-03	0.064
S6 pS235 S236	6.25	2.6E-03	0.064

Correlation statistics between changes in biopotential and protein expression between cancerous and non-cancerous samples, respectively (n = 10). The tumour cellularity difference between cancerous and non-cancerous samples are also included in this model. Linear regression analysis was performed to generate these statistics, including the log2 fold change (logFC) of the trend observed, the p-statistic and adjusted p-statistic. Top 10 most significant trends are shown, with CDK1 being the only statistically significant observation.

From the RPPA data of the paired HGSOC tissue samples, further linear regression analysis was performed to identify any associations in protein expression changes between paired samples and patient outcome. A number of proteins were identified to be statistically significantly altered (Supplementary Table S1). Only lactate dehydrogenase A (LDHA) showed a clear differential expression related to patient outcome, with high levels of LDHA at the onset of the disease being associated with a significantly higher risk of future relapse and poor outcome (Supplementary Figure S5).

### Lower biopotential is associated with increased lactate/glucose ratio

**Figure 5:**
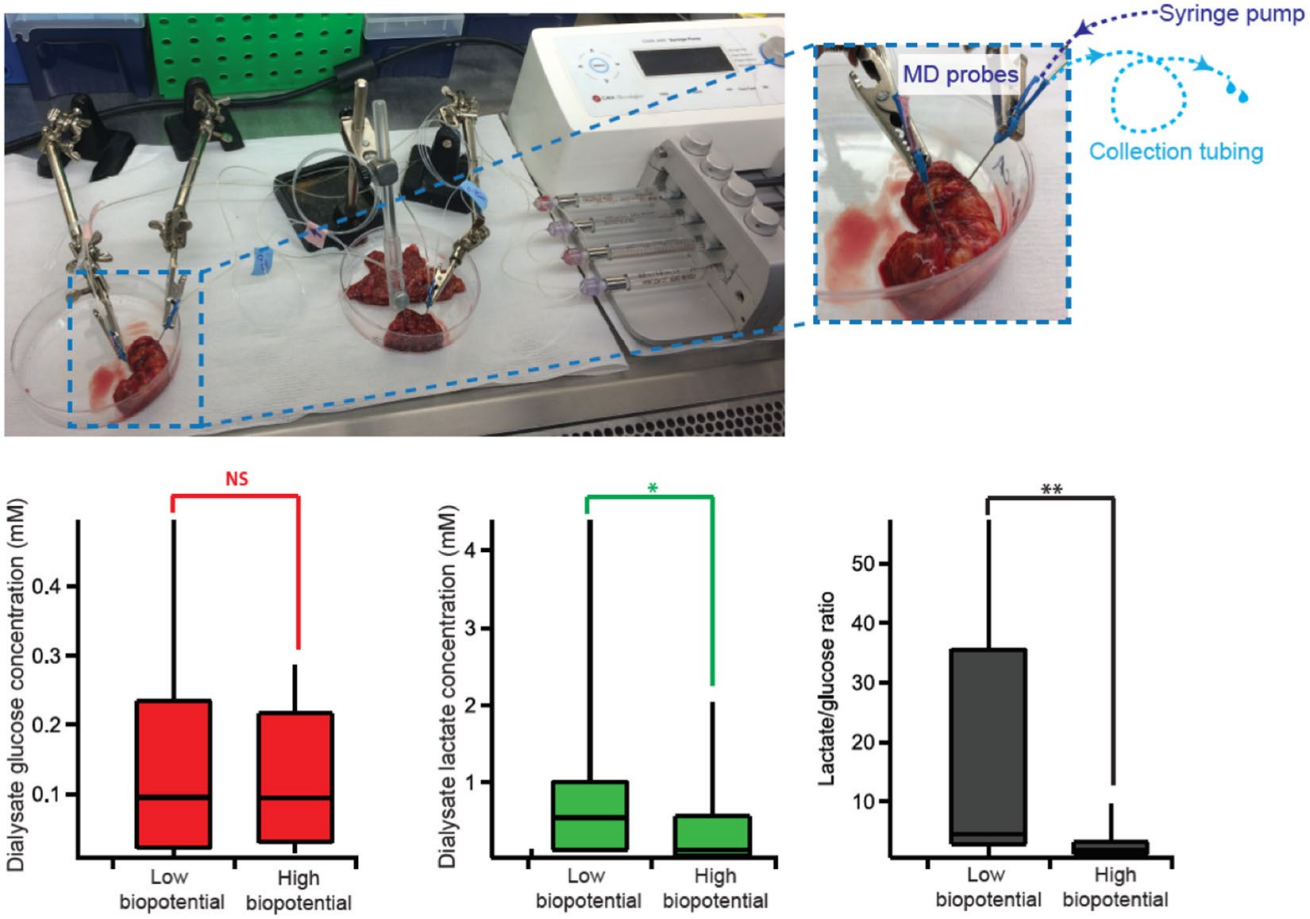
Laboratory setup for dialysate collection. (**a**) Two probes are inserted into non-cancerous tissue (left) and cancerous tissue (right) and perfused simultaneously using a syringe pump. The dialysate is collected in 0.75 m lengths of collection tubing. (**b**) Box and whisker diagrams comparing dialysate glucose levels (red), lactate levels (green) and lactate/glucose ratio (black) for paired omentum tissue with low and high biopotential (n = 12). Statistics tested using two-tail Wilcoxon sign rank test.

As cancerous cells often exhibit anaerobic metabolism, in some of our paired *ex vivo* omentum samples dialysate levels of glucose and lactate were measured at the same time as the biopotential was measured. The levels of these metabolites and the corresponding lactate/glucose ratio, which is often found to be a more sensitive marker than either metabolite alone^19^, for these paired omentum samples are shown in Fig. 5b. The results indicate that cancerous omentum has significantly higher lactate levels than its paired non-cancerous appearing tissue (p < 0.05). This is also reflected in the lactate/glucose ratio, which is significantly higher in cancerous omentum (p < 0.01).

## Discussion

Our study demonstrates, for the first time, a significant predictive value of the bioelectrical properties of cancerous tissue for higher risk of recurrence, poor clinical outcome as well as advanced stage in patients with epithelial ovarian cancer. In an era where ground-breaking novel bioengineering advances are finding their way into many aspects of medical care, current bioengineering advances have focused on rapid diagnostics and identification of tumour margins such as the iKnife^20^, the MasSpec Pen^21^, and fiber pH probe^22^. In this study, we have established a novel bioengineering approach which could reliably inform on patients’ clinical and surgical outcome and guide surgical decision-making, as well as distinguishing between cancerous and non-cancerous tissue. Especially in cancer surgery, the development of validated algorithms that could guide personalised surgical and clinical management is warranted in an effort towards a more individualised care approach. From our approach profiling the bioelectrical and metabolic properties of cancer tissue correlated with validated biomarkers of outcome, there is a potential opportunity to use this information to develop a pre-operative algorithm that could help tailor surgical radicality directing an ultra-radical approach to patients that will benefit most. Thus, sparing those patients who will fail radical treatment and subsequently relapse early, an unnecessary surgical morbidity and direct them towards alternative therapeutic principles.

From a biomedical engineering standpoint, we have demonstrated that it is possible: (a) to conceive novel methods to measure precisely biophysical properties (tissue biopotential) and (b) engineer biochemical tissue signature (energy metabolites) measurement methods even in the difficult circumstances of excised tissue from an ongoing operation. To achieve this, it was necessary to develop novel sampling probes and controlling circuitry and then translate them to a clinical environment. We have shown that our measurement devices are practical and produce results with potential clinical value.

We observed consistent significantly different biopotential values in the cancerous tissue compared to the paired non-cancerous tissue; this was the case in all patients analysed. In an effort to identify what factors could influence bioelectrical properties, we demonstrated that biopotential measurements are influenced by matrix stiffness and cell density using *in vitro* agarose and 3D collagen models. Several studies have demonstrated that extra-cellular matrix stiffening promotes the progression of many cancers^23^, and a recent study examining the evolving tumour microenviroment of EOC omental metastasis suggests a close association of tissue matrix stiffness with disease progression^24^. In the follow-up validation of our current feasibility study we will aim to correlate tissue biopotential measurements with corresponding tissue stiffness measurements, to confirm the relationship between matrix stiffness and biopotential. We identified a positive correlation between increasing tumour load and biopotential levels in cancerous tissues, and a negative association between low biopotential levels and increasing amount of stromal tissue present. The role of the stromal compartment in tumours has been shown to be indicative of a poorer outcome in cancers including EOC^25,26^. Moreover, the size of sample or tissue measured did not influence subsequent biopotential readings, demonstrating that the tungsten measurement electrode only detected the biopotential signal in its immediate vicinity.

In relation to clinical parameters, we could demonstrate a significant correlation between biopotential levels of cancerous omentum with tumour stage and clinical outcome. High biopotential levels in the cancerous omentum were significantly associated with advanced stage IV tumour. Furthermore, when stratifying at median biopotential over all the omental tumours assessed, levels below the median were associated with a poorer progression-free survival for all EOC histological subtypes, and the most prevalent HGSOC subtype also. Proteomic analysis of a subset of HGSOC cases in our cohort revealed the most common pathways associated with changes in biopotential between cancerous and non-cancerous tissues were apoptosis related pathways, and altered CDK1 expression significantly correlated with changes in biopotential. It is known that deregulation of pro- and anti-apoptotic pathways is a key factor in the onset and maintenance of chemotherapy resistance in ovarian cancer, and many genes involved in apoptosis are aberrantly regulated in ovarian cancer. Overexpression of CDK1 has been associated with several cancers, in particular in EOC increased expression of CDK1 correlated with poor prognosis^27,28^, and may also be a valuable biomarker for EOC to incorporate into a potential prognostic algorithm.

There was also a clear positive clinical correlation in the proteomic analysis of the paired HGS cancerous versus non-cancerous samples with increased LDHA expression being associated with a significantly higher risk of future relapse and poor outcome. One of the most fundamental steps in the process of aerobic glycolysis is the conversion of pyruvate to lactate in cytosol by the NADH-dependent enzyme, lactate dehydrogenase (LDH)^29^. LDHA overexpression has also been observed in different cancer types including ovarian cancer subtypes and shown to be a potential feedback activator of hypoxia inducible factor in ovarian cancer cell lines^30,31^. Furthermore, our metabolic profiling for lactate and glucose levels demonstrated that higher lactate and lower glucose levels coincided with lower biopotential readings in the cancerous tissue compared with the paired non-cancerous appearing tissue. This is likely due to the inherently hypoxic tumour environment leading to elevated levels of lactate in cancerous tissue but also due to the higher energy consumption and hence glucose consumption by the cancerous tissue. These preliminary metabolite results are promising and suggest that levels of metabolites along with potential tumour biomarkers such as LDHA could contribute to a panel of markers that could provide insight into tumour biology, associated with corresponding biopotential profiles.

This type of approach and deployment of bioengineering principles and techniques in ovarian cancer surgery is unique. For that reason, we had to adapt currently available bioengineering tools to the measurements of omentum tissue following dissection from oncologic cytoreductive surgeries and develop new techniques and principles. We have intentionally chosen a tungsten working electrode for the biopotential measurements because of its properties; it is hard, chemically inert, does not oxidize or corrode easily, and is resistant to attack from acids and alkalis^32^, rendering it ideal for voltage measurements in tissue samples. A double junction Ag|AgCl reference electrode was preferred to avoid contamination of the results by junction potentials, the relative convenience of manufacturing, its high reproducibility and the good stability of its potential^33^. A high-performance instrumentation amplifier was preferred because it removes the common-mode noise from the input signal. Thus, the output amplified signal was less susceptible to noise. Another advantage of using an instrumentation amplifier was that it offers a very high differential mode input impedance for efficient interfacing with the reference electrode. The metabolic measurements were more challenging; even though the microdialysis sampling has allowed us to gather preliminary metabolite data from human tissue samples, in some cases it was technically very difficult to obtain reliable measurements from non-cancerous omentum tissue as the tissue was very thin and compliant, leading to issues inserting the probe completely without damaging the membrane. These dialysate metabolite readings obtained have provided us with a range of expected metabolite levels in cancerous and non-cancerous tissue, guiding future biosensor development.

Our approach, while still in its preliminary stages and relying on bioengineering principles not applied in this manner previously, has potentially transformative implications on clinical practice. Our next aim is to validate these findings in a large prospective cohort, to ascertain the feasibility that clinicians could use the bioelectrical properties of cancerous tissue to reliably predict outcome of their patients and hence tailor their treatment and individualise intended radicality. Furthermore, in an effort to make the actual tissue measurements less challenging and more user friendly for clinicians, we are in the process of developing a microprobe pen-like device which will miniaturise and incorporate the technology presented here to detect biopotential and metabolites in real time and hence be able to generate in the theatre a signature predictive of outcome either *in vivo* or *ex vivo* after tumour excision or even from a biopsy of cancerous tissue. On the basis of the biopotential signature obtained, clinicians would be able to allocate patients towards a standard versus ultra-radical surgical approach, performing the latter only on those who will have a true survival benefit when balanced with the resultant iatrogenic morbidity. This research approach will significantly contribute towards personalising surgical care, a large area of highly unmet need for patients with advanced EOC, while presenting innovative bioengineering technology which could be applied to other cancer types in the future.

## Methods

### Intra-operative collection of patient samples and clinical follow up

At the time of cytoreductive surgery for advanced primary EOC, macroscopically cancerous omentum and paired macroscopically non-cancerous omentum tissues were collected and immediately transferred to the laboratory for *ex vivo* biopotential measurements and further analysis. All surgical and clinical patient and tumour related data were prospectively collected (Table 1). The project was performed under the Hammersmith and Queen Charlotte’s and Chelsea Research Ethics Committee approval 05/QO406/178, and supplied by the Imperial College Healthcare NHS Trust Tissue Bank, following full informed patient consent. All methods were performed in accordance with the relevant guidelines and regulations. Inclusion criteria for our study comprised patients undergoing primary maximal effort cytoreductive surgery for advanced EOC, FIGO stage III-IV, chemotherapy-naïve, with no previous malignancies. Patient samples were exclusion from the study if the patients had received neo-adjuvant chemotherapy, or were non-EOC or non-ovarian histotype.

Patients were regularly evaluated at the end of their surgical and systemic treatment for evidence of disease recurrence. Clinical history, examination and CA-125 (if the pre-operative value was elevated) were performed every 3 months for the first 2 years and then 6-monthly. A CT/MRI-scan was ordered if the above examinations revealed any pathology. Isolated CA-125 elevation was not regarded as a recurrence.

### Biopotential measurement collection

Paired macroscopically-appearing cancerous and non-cancerous omentum tissues of all evaluated patients were assessed for their bioelectrical properties. Biopotential was measured using an experimental setup comprised of an electrochemical cell consisting of two half cells, a tungsten working electrode, a silver-silver chloride (Ag|AgCl) double junction reference electrode, an instrumentation amplifier, an RPMI 1640 cell culture medium (Life Technologies, Carlsbad, CA), and one ml pipette tips. The electrode (UEWSHGSE3N1M from FHC) is made up of two regions (Supplementary Figure S6b). The first region is the tungsten material, which is fully insulated with epoxylite insulation. The shank diameter of this region is 500 µm. Only the final tip, which is 120 µm in length with a diameter of less than 1 µm, is exposed (conductive) to be able to take voltage measurements. The final taper angle is 10°−15°. The second region (gold part) is conductive and is used to connect the electrode with the electronic equipment.

As illustrated in Fig. 1b, the tissue sample is placed in a pipette tip, partly immersed in media in a beaker. The bottom of the pipette tip is open so that the tissue is in direct contact with the media. The tip of the tungsten electrode pierces the surface of the tissue without making contact with the media. This ensures that the voltage difference measurement is taken from the interior of the tissue sample only. The Ag|AgCl electrode is also placed in media inside a second pipette tip forming a double-junction reference electrode immersed in the media in the beaker. A double-junction reference electrode was preferred because it allows the chloride (Cl−) contained in the Ag|AgCl reference electrode to slowly mix with the media in the pipette tip and not with the tissue. This prevents contamination of the tissue by chloride and contamination of the Ag|AgCl reference electrode by the blood/liquid coming from the tissue. Fresh media is used in each experiment.

The negative and positive terminals of the instrumentation amplifier were connected to the Ag|AgCl reference electrode and the tungsten electrode, respectively. The instrumentation amplifier recorded and amplified the potential difference between the two electrodes, while the electrochemical cell had to be in equilibrium before taking biopotential measurements. The output of the instrumentation amplifier was connected to a data acquisition system (PowerLab, ADInstruments), which converted the analogue output from the instrumentation amplifier into a digital signal. This signal was visualized on a computer for further analysis. More details on the adapted method can be found in^14^. The biopotential of each tissue sample was measured over a 2-minute period per measurement in 3 different areas of each tissue collected, and data was normalised to the reference media biopotential measurement.

### Determination of tumour cellularity

In order to associate biopotential levels with tumour and stromal cellularity following biopotential measurement, measured tissue pieces were retained for histological assessment using common ovarian cancer tumour markers and determination of a tumour cellularity score. Following collection of biopotential and metabolite dialysate, tissue samples were snap frozen and stored at −80 °C prior to histological processing. Tissue samples were sectioned using a Bright Cryomicrotome and 10 μm sections were collected. Haematoxylin and eosin staining was carried out for each sample. For tumour and stroma confirmation, a selection of tumour (Pax8 – Abcam cat. no. 189249 and WT1 – Abcam cat. no 89901) and mesothelial cell markers (Calretinin – Dako, cat. no M7245) were used. All sections were fixed with 4% PFA (ChemCruz, sc-281692) for 10 minutes at room temperature (RT) and washed 3 times, every 5 minutes with 1x tris buffered saline (TBS). Pax8 staining required an additional step of antigen retrieval with 10 mM sodium citrate buffer pH8.5 for 40 minutes. All sections were treated with an endogenous peroxidase blocking solution (70% methanol, 28% deionized water and 2% hydrogen peroxide) for 5 minutes, washed 3 × 5 minutes with 1x TBS and blocked/permeabilised with a solution of 6% goat serum and 0.5% Triton X-100 in 1x TBS for 1 hour. Primary antibodies were incubated overnight at 4 °C at 1:50 Pax8, 1:200 WT-1, and 1:50 Calretinin, in 1x TBS with 6% goat serum and 0.1% Triton X-100. Sections were washed with 1x TBS 3 × 5 minutes, and incubated in the corresponding secondary antibodies: 1:200 biotinylated anti-rabbit IgG (Vector lab BA-1000) or biotinylated anti-mouse IgG (Vector lab BA-9200) for 30 minutes at RT. Sections were washed with 1x TBS and the avidin-biotin complex was developed with the Vectastain ABC kit (Vector lab PK-6100) for 30 minutes at RT, followed by 1x PBS for 5 minutes. Peroxidase substrate (ImmPACT DAB Peroxidase substrate, Vector Lab SK-4105) was added for 30 seconds for sections stained with WT1 and Calretinin, and 40 seconds for sections stained with Pax8. All sections were counterstained with haematoxylin (LAMB, 230-D) and mounted in DPX (Sigma, 06522). All sections were scanned using a Zeiss AxioScan Z1. The images obtained were analyzed with QuPath software^34^ to determine the tumour cellularity of each tissue sample measured for biopotential and metabolites.

### Tumour protein lysate preparation

Ten frozen 30 μm sections of tissue were collected by cryotomy from two paired cancerous and non-cancerous omentum tissues from 10 cases from our sample cohort, all high grade serous ovarian cancer (HGSOC), and mechanically lysed in cold RPPA lysis buffer 1% Triton X-100, 50 mM HEPES, pH 7.4, 150 mM NaCl, 1.5 mM MgCl_2_, 1 mM EGTA, 100 mM NaF, 10 mM Na_4_P_2_O_7_, 10% glycerol containing freshly added protease (Roche Applied Science, 05056489001) and phosphatase inhibitors (VWR, 524625)), lysates were incubated on ice for 30 minutes and centrifuged at 14000 rpm for 10 minutes at 4 °C. The supernatants were retained and stored at −80 °C. Protein concentrations were determined by BCA assay (Thermo Scientific). Lysates were shipped to the RPPA facility, MD Anderson Cancer Center, USA and analysed as previously described^35^.

### Dialysate collection for metabolite measurement

In order to measure the metabolites of the tissues and correlate it with the biopotential measurements, two CMA 12 microdialysis probes (Linton Instruments, 4 mm membrane length) were inserted into each sample of either cancerous or non-cancerous omentum tissue. The CMA probes were chosen because they were rigid, even so it was necessary to use a 21-gauge hypodermic needle to make a small hole in the tissue before inserting the smaller diameter microdialysis probe to avoid damage to the membrane. The probes were positioned close together and were perfused at 1 μl/min with T1 perfusion solution (2.3 mM calcium chloride, 147 mM sodium chloride, 4 mM potassium chloride). The outlet of each probe was connected to a 0.75 m length of coiled Portex fine-bore storage tubing (0.4 mm inner diameter, Smiths Medical, UK)^36^ in a sterile tissue culture hood, as shown in Figure 5a. The storage tubing was primed with T1 solution and was labelled to record the direction of flow. After 30–40 min of dialysate collection, the sample tubes were sealed to avoid losing the sample and frozen until analysis for levels of glucose and lactate using the microfluidic biosensor system.

### Microfluidic biosensor system

To analyse each sample, the stored dialysate was pumped through the analysis system, effectively ‘replaying’ the liquid stream as if it were flowing in real time. Our custom-built microfluidic biosensor system has been described previously^16,17^. Briefly, glucose and lactate biosensors were fabricated using combined needle electrodes^37^ and functionalized in three layers^38,39^ and were positioned in a poly(dimethylsiloxane) (PDMS) microfluidic chip^40^. The thickness of the outer diffusion-limiting layer can be varied to control the operational range and sensitivity of the biosensors.

### Statistical analysis

All statistical analysis was done using R 3.3.1. For all analysis, three raw biopotential readings were measured and were normalized to the reference media control. The average value of the three replicates was then calculated and referred below as biopotential readings or biopotential. Differential biopotential by stage was compared by two sample t-test with unequal variances. Survival analysis including the Kaplan-Meier plots and Cox regression was performed using ‘survival’ package. Log-rank test was applied to compare the survival distribution of the two patient groups split at median biopotential readings. Cox regression of the dichotomized patient groups with progression-free survival was performed to confirm the result of the log-rank test. Correlation between tumour, stromal or adipose content with biopotential was measured by Spearman’s correlation coefficient (r) and t-test.

## Electronic supplementary material

Supplementary Information

## Data Availability

The datasets generated during and/or analysed during the current study are available from the corresponding author on reasonable request.
