# Supplementary material for: Clinical value of bioelectrical properties of cancerous tissue in advanced epithelial ovarian cancer patients
Source: Sci Rep. 2018 Oct 2;8:14695. doi: 10.1038/s41598-018-32720-8 (PMC6168525; doi:10.1038/s41598-018-32720-8)
Supplement: Supplementary file 1 — Supplementary Information [file 41598_2018_32720_MOESM1_ESM.docx]

**Clinical value of bioelectrical properties of cancerous tissue in advanced epithelial ovarian cancer patients.**

Paula Cunnea^1^, Tommy Gorgy^1^, Konstantinos Petkos^2^, Sally A.N. Gowers^2^, Haonan Lu^1^, Cristina Morera^1^, Wen Wu^2^, Phillip Lawton^1^, Katherine Nixon^1^, Chi Leng Leong^2^, Flavia Sorbi^1,3^, Lavinia Domenici^1,4^, Andrew Paterson^1^, Ed Curry^1^, Hani Gabra^1,5^, Martyn G. Boutelle^2^, Emmanuel M. Drakakis^2^, Christina Fotopoulou^1^*.

**Supplementary Materials and Methods**

***Materials and reagents***

Glucose oxidase (GOx) from Aspergillus niger, lactate oxidase (LOx) from Aerococcus viridians were purchased from Sekisui Diagnostics. All other reagents were obtained from Sigma-Aldrich. Portex fine-bore polyethylene tubing was purchased from Smiths Medical, UK. RPMI media and supplements, and RPPA buffer components were purchased from Sigma-Aldrich. SKOV3 ovarian cancer cell lines were purchased from ATCC and maintained in RPMI 1640 plus 10% foetal bovine serum, L-glutamine and penicillin-streptomycin at 37ºC and 5% CO_2_, and mycoplasma tested at frequent intervals. Cells at low passage were used for each experiment.

***In vitro agarose gel biopotential measurements***

In order to understand which factors influence the measured biopotential properties, experiments were conducted measuring biopotential with a) different sizes of measured tissue to assess whether size of tissue matters, b) agarose gels of different concentrations (3%-7%) by dissolving the appropriate amount of agarose powder (Fisher Bioreagents) per concentration in RPMI 1640 media, heated and allowed to cool to solidify to assess whether matrix density has an impact and c) different numbers of SKOV3 ovarian cancer cells (8 x 10^6^, 12 x 10^6^ and 16 x 10^6^) suspended in a collagen mix containing 80% Rat tail collagen I (Corning) to assess whether tumour cell concentration has a bioelectrical impact.

***Bioinformatic analysis of RPPA data***

Pathway enrichment analysis was used to determine the most commonly up-regulated or down-regulated pathways altered when comparing protein expression changes related to changes in biopotential between non-cancerous and cancerous tissues. Bioinformatic analyses were carried out using R v3.1.0 (https://www.r-project.org/). Linear regression was performed using the lmFit function within the limma package. An average change in biopotential for each patient was obtained by subtracting the media normalized biopotential for cancerous samples from the respective non-cancerous samples for each patient (n=9). Using linear regression, this was compared to the average log10 fold change of proteins between cancerous and non-cancerous samples. The average cellularity difference of the cancerous vs non-cancerous samples from each individual patient was added as the intercept in the design matrix alongside the change in biopotential. This was calculated by averaging the two samples of each type per patient and subtracting the cancerous cellularity from the non-cancerous. Statistics obtained include the log10 fold change and the p statistic of the trend. Adjustments to the p values were made using the Benjamini-Hochberg method and adjusted p values of <0.05 were considered significant.

Pathway information for analysis was obtained from Consensus Path DB (http://cpdb.molgen.mpg.de/), and pathways were filtered to only those where at least two pathway components were represented by probes on the protein array. Changes in protein expression between cancerous and non-cancerous samples were ranked based upon the log10 fold change of the trend. The geneSetTest function in the limma library was used to perform pathway enrichment analysis. P statistics were obtained and adjusted using the Benjamini-Hochberg method and adjusted p values of <0.05 were considered significant.

Linear regression, as performed using the lmFit function within the limma package, was used to find associations in protein expression with whether the patient was in remission (n=7) or have had poor outcomes (n=3). Protein expression was determined as the average log10 fold change of proteins between cancerous and non-cancerous samples. This was also separately performed with the change in biopotential between cancerous and non-cancerous samples as the intercept for the model (poor outcomes n=3; remission n=6). An average change in biopotential for each patient was obtained by subtracting the media normalized biopotential for cancerous samples from the respective non-cancerous samples for each patient. Statistics obtained from these analyses include the log10 fold change and the p statistic of the trend. P values were corrected for multiple testing using the Benjamini-Hochberg method and adjusted p values of <0.05 were considered significant.

**Supplementary Figure and Table legends:**

**Supplementary Figure S1**: Biopotential measurements are not influenced by tissue sample size. Biopotential readings were measured from cancerous tissues while dissected into smaller pieces (width or depth), no differences were observed between tissues differing in size (n=3).

**Supplementary Figure S1**

**Supplementary Figure S2:** Immunohistochemistry staining for Pax8 and WT1 in frozen sections of paired macroscopically cancerous and non-cancerous omentum tissue from one case of HGSOC. The corresponding haematoxylin and eosin (H&E) section is also displayed. Sections were scanned using AxioScan at 10X magnification (larger image), smaller inset images depict positive staining for Pax8 and WT1. Small areas of micrometastasis of tumour cells in the non-cancerous tissues is observed. NCO: Non-cancerous omentum, CO: cancerous omentum.


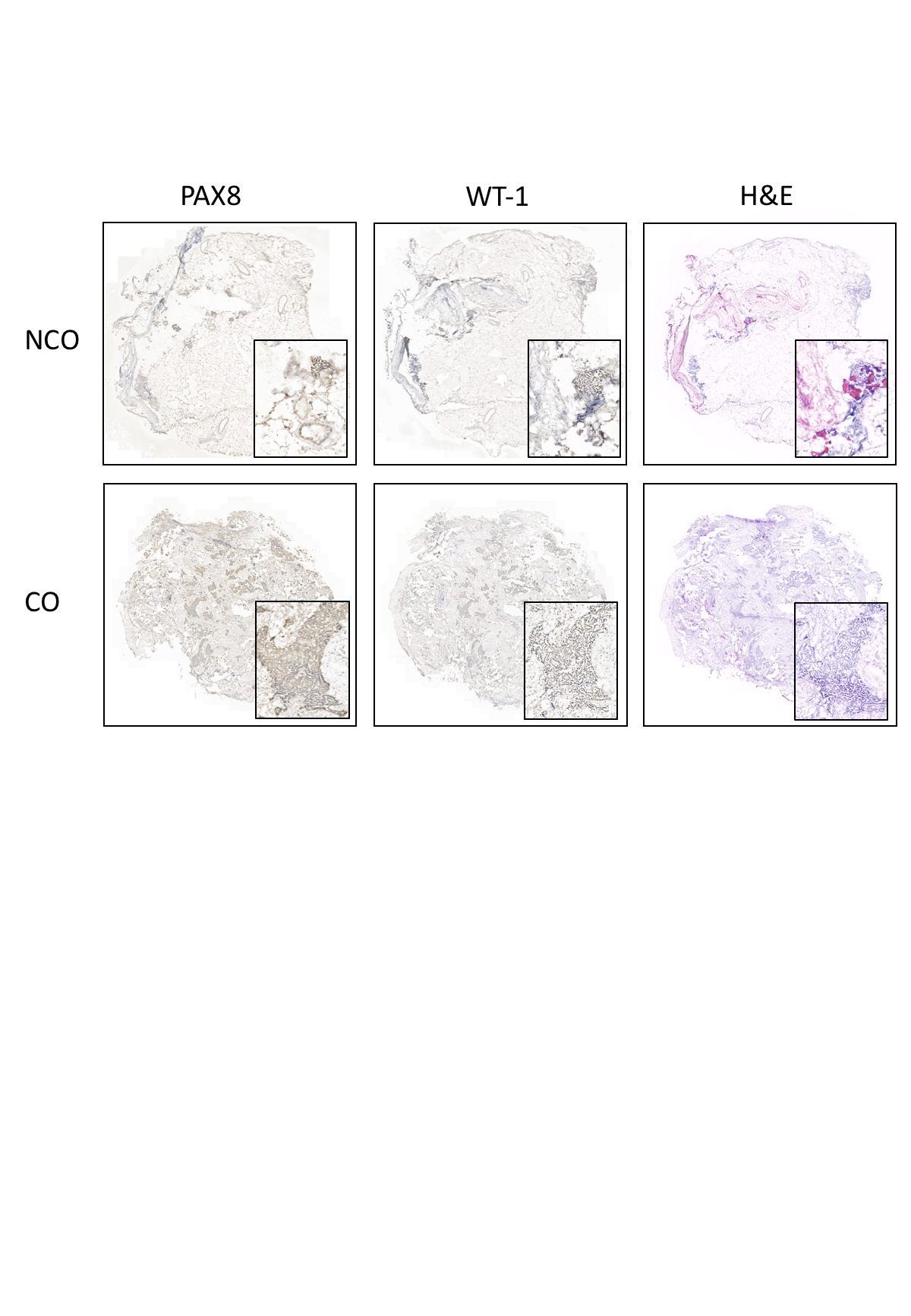


**Supplementary Figure S3:** a) Tumour content and biopotential is not correlated in non-cancerous tissue. b) Stromal content and biopotential is not strongly correlated in non-cancerous tissue. c) Adipocytes content and biopotential is not strongly correlated in cancerous tissue.

**a**

**b**

**c**

**Supplementary Figure S4:** Matrix density and cell rate influence biopotential levels. a) Biopotential readings were captured from collagen matrices established with different SKOV3 cell numbers, decreasing biopotential readings were observed in matrices with higher cell numbers (n=3). b) Agarose gels of different densities were constructed and biopotential readings captured, showing decreasing biopotential levels in increasing agarose concentrations (n=3).

**Supplementary Figure S4**

**a**

**b**

**Supplementary Figure S5:** LDHA protein expression change between cancerous and non-cancerous samples for patients that are in remission (n=7) and those that have had poor outcomes (n=3). Expression changes are statistically significant (p=0.014).

**Supplementary Figure S5**


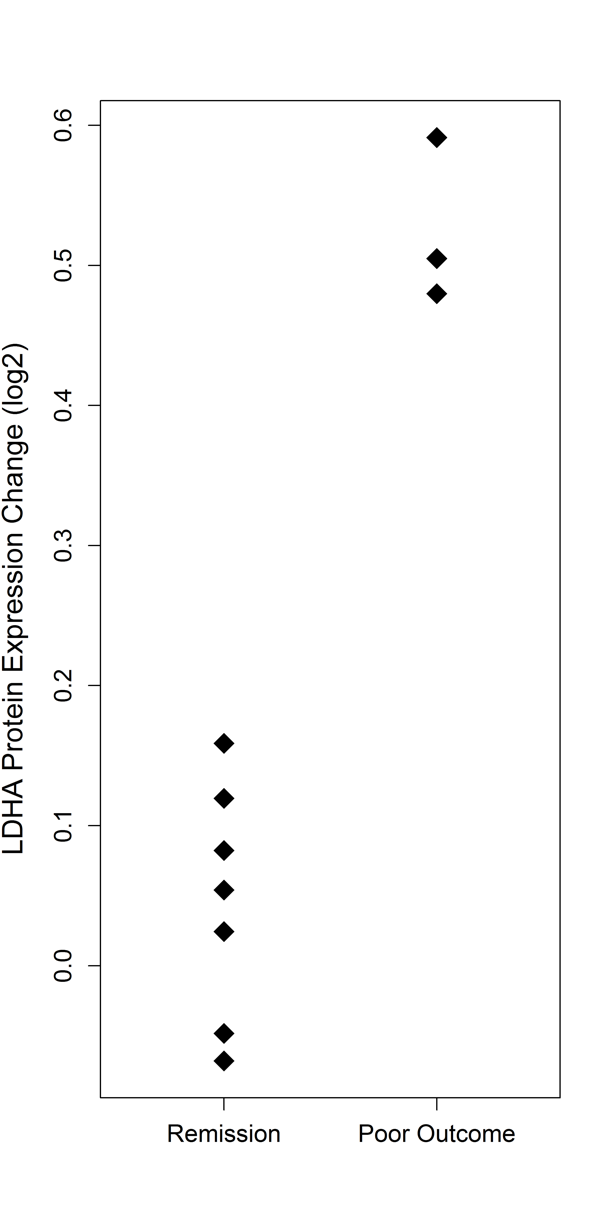


**Supplementary Figure S6:** a) Graphical representation of the analog front end used for the recording of biopotential signals. (A) The signals coming from the tungsten and the Ag|AgCl reference electrode are subtracted and amplified with a gain of 10 by the instrumentation amplifier (INA), (B) buffered by an operational amplifier (OPA), (C) filtered by an analog low pass filter (LPF) at 10 Hz, converted to digital by an analog to digital converter (ADC) and depicted on the computer using Labchart software. b) Tungsten working electrode (UEWSHGSEN1M from FHC) used for biopotential tissue measurements. The tungsten part of the electrode (black section) is fully insulated apart from the tip which is 120 μm in length, less than 1 μm in diameter and is characterized by a taper angle of 10º-15º. The conductive tip of the electrode allows for the collection of the tissue voltage measurements. The second part of the electrode (golden part) is conductive and enables the connection of the electrode with the electronic equipment.

**Supplementary Figure S6a**


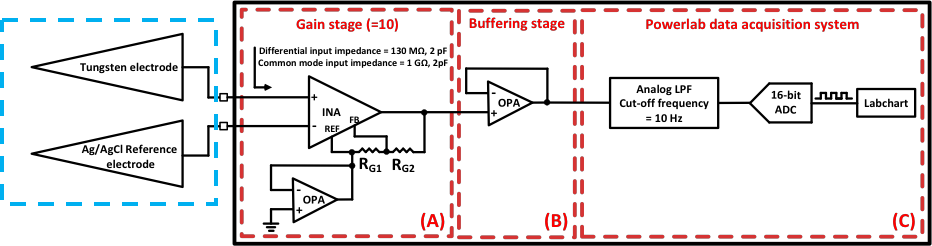


**Supplementary Figure S6b**


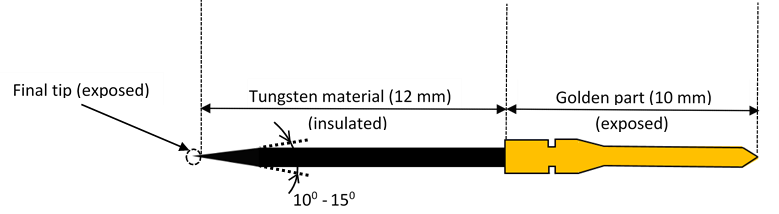


**Supplementary Table S1:** Linear regression was performed to identify correlations between protein expression changes between cancerous and non-cancerous samples, and the status of patient outcome. Statistics shown from this analysis are the log2 fold change (logFC) of the trend observed, the p-statistic and adjusted p-statistic, with the top 10 most significant genes shown in the table. Only LDHA demonstrates differential expression between patients with poor outcome, and patients that remain in remission.

| Protein Probe | logFC | P Value | Adjusted P Value |
| --- | --- | --- | --- |
| LDHA | 0.48 | 4.8E-05 | 0.014 |
| Myosin IIa pS1943 | 0.93 | 3.0E-03 | 0.46 |
| ATRX | -0.34 | 6.0E-03 | 0.57 |
| PRAS40 | 0.42 | 9.2E-03 | 0.57 |
| SOD1 | 0.22 | 9.6E-03 | 0.57 |
| Gys | 0.29 | 1.8E-02 | 0.75 |
| GAPDH | 1.02 | 2.0E-02 | 0.75 |
| Rab25 | 0.57 | 2.2E-02 | 0.75 |
| Transglutaminase | 0.24 | 2.3E-02 | 0.75 |
| Stat5a | -0.31 | 2.9E-02 | 0.88 |
